# miR-22 suppresses DNA ligase III addiction in multiple myeloma

**DOI:** 10.1038/s41375-018-0238-2

**Published:** 2018-08-17

**Authors:** Daniele Caracciolo, Maria Teresa Di Martino, Nicola Amodio, Eugenio Morelli, Martina Montesano, Cirino Botta, Francesca Scionti, Daniela Talarico, Emanuela Altomare, Maria Eugenia Gallo Cantafio, Valeria Zuccalà, Lorenza Maltese, Katia Todoerti, Marco Rossi, Mariamena Arbitrio, Antonino Neri, Pierosandro Tagliaferri, Pierfrancesco Tassone

**Affiliations:** 1 Department of Experimental and Clinical Medicine, Magna Græcia University, Campus Salvatore Venuta, Catanzaro, Italy; 2 Pathology Unit, Pugliese-Ciaccio Hospital, Catanzaro, Italy; 3 Department of Oncology and Hemato-oncology, University of Milan, and Hematology, Fondazione Cà Granda IRCCS Policlinico, Milan, Italy; 4 Sbarro Institute for Cancer Research and Molecular Medicine, Center for Biotechnology, College of Science and Technology, Temple University, Philadelphia, PA USA

**Keywords:** Apoptosis, Myeloma

## Abstract

Multiple myeloma (MM) is a hematologic malignancy characterized by high genomic instability. Here we provide evidence that hyper-activation of DNA ligase III (LIG3) is crucial for genomic instability and survival of MM cells. LIG3 mRNA expression in MM patients correlates with shorter survival and even increases with more advanced stage of disease. Knockdown of LIG3 impairs MM cells viability in vitro and in vivo, suggesting that neoplastic plasmacells are dependent on LIG3-driven repair. To investigate the mechanisms involved in LIG3 expression, we investigated the post-transcriptional regulation. We identified miR-22-3p as effective negative regulator of LIG3 in MM. Enforced expression of miR-22 in MM cells downregulated LIG3 protein, which in turn increased DNA damage inhibiting in vitro and in vivo cell growth. Taken together, our findings demonstrate that myeloma cells are addicted to LIG3, which can be effectively inhibited by miR-22, promoting a novel axis of genome stability regulation.

## Introduction

Hallmarks of cancer are produced by progressive genomic aberrations that finally confer selective growth advantage to cancer cells [1]. Multiple myeloma (MM) is still an incurable hematologic malignancy characterized by deep genomic instability [2], which leads to abnormal proliferation of malignant plasma cells harboring several genetic karyotype aberrations, in the absence of universal driver mutations [3, 4]. Although the molecular bases of these events are largely unknown, it is possible to speculate that alterations in one or more components of the DNA-damage repair machinery may be involved in disease development and progression [5, 6].

Response to DNA damage relies on large number of proteins and different classes of enzymes, which converge to the end-joining of DNA strands, catalyzed by DNA ligases. There are three human genes encoding DNA ligases [7]. LIG1 gene encodes DNA ligase I (LigI), which predominantly operates in DNA replication [8], with minor role in excision repair process (BER and NER), and alternative non homologous end joining (Alt-NHEJ) repair. LIG4 gene encodes DNA ligase IV (LigIV), which catalyzes the last step of canonical-NHEJ repair pathway [9]. Unlike LIG1 and LIG4, the LIG3 gene encodes multiple DNA ligase isoforms. Indeed, alternative translation initiation generates two different isoform of DNA ligase IIIα (LigIIIα) [10]: (a) nuclear LigIIIα, predominantly involved in excision repair process and Alt-NHEJ repair [11]; (b) mitochondrial LigIIIα, which is the DNA ligase involved in mitochondrial genome replication and repair [12]. In addition, a germ cell-specific alternative splicing mechanism of LIG3 generates DNA ligase III β (LigIIIβ), which has been hypothesized to be involved in meiotic recombination and/or DNA repair in haploid sperm [13].

In this study, we aimed to identify the potential role of DNA ligases as promoters of genomic instability and survival in MM, and we investigated the involved upstream regulation mechanisms. To this aim, we focused on microRNAs (miRNAs), given their relevant role in the regulation of DNA repair machinery [14], largely unknown in MM.

miRNAs are small non-coding RNAs of 19–25 nucleotides, which regulate the gene expression by degrading or inhibiting the translation of target mRNAs, primarily via base pairing to partially or fully complementary sites in the 3′UTR [15]. Targeting deregulated miRNAs in cancer cells is rising as a novel therapeutic option [16, 17] for a variety of solid tumors. miR-34a and miR-16 formulated mimics have already reached the phase I clinical evaluation demonstrating antitumor activity in a subset of patients with refractory advanced solid tumors [18, 19]. Deep dysregulation of miRNAs has been widely described in hematologic malignancies, including MM [20–32], where new miRNA-based therapeutics are presently in advanced phases of pre-clinical investigation and are approaching to clinical translation [33]. In MM, miRNA-replacement by synthetic miRNA mimics may offer a therapeutic opportunity [34] to simultaneously restore multiple anti-proliferative and pro-apoptotic pathways. Importantly, miRNAs can target several components of DNA damage response (DDR), and therefore modulate mechanisms involved in preserving genomic integrity [35–37].

Here we identified LIG3 as crucial node of DNA-repair machinery and survival of MM cells, and we characterized miR-22 as negative regulator of DNA ligase III addiction in MM.

## Materials and methods

For a more detailed description of the methods used, see supplementary methods (available on the online version of paper).

### Cell lines and primary tumor specimens

Cell lines were obtained from the ATCC or kindly provided by sources indicated in Supplementary Methods.

### Analysis of cell viability and apoptosis

Cell viability was analyzed by Cell Titer-Glo assay (CTG; Promega, Madison, WI, USA); apoptosis was evaluated by flow cytometric (Attune NxT Flow cytometer, Thermo Fisher Scientific) analysis following Annexin V-7AAD staining (BD Pharmingen).

### RNA extraction and quantitative real-time-PCR

Total RNA extraction from MM cells and qRT-PCR were performed as previously described [25]. Procedures are in Supplementary Methods.

### Plasmids, transfection, and transduction of MM cells

Plasmids used and related procedures are reported in Supplementary Methods.

### In vivo studies

The MM xenograft models used are described in Supplementary methods.

### Statistical analysis

Each experiment was performed at least three times and values are reported as means ± SD. Comparisons between groups were made with Student’s *t*-test, while statistical significance of differences among multiple groups was determined by GraphPad software (www.graphpad.com). Graphs were obtained using Graphpad Prism version 6.0. *p* value of less than 0.05 was accepted as statistically significant. The synergistic index was determined as previously described [38].

## Results

### High LIG3 expression occurs in MM and predicts poor prognosis

**Fig. 1:**
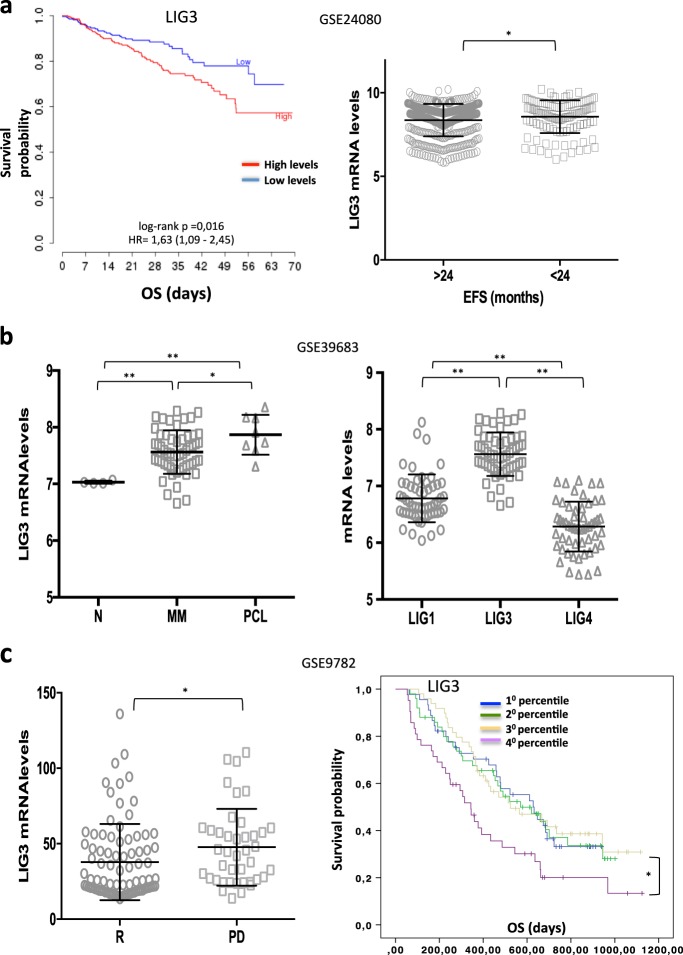
High LIG3 levels are associated with poor prognosis in multiple myeloma patients. **a** Data obtained from GSE24080 dataset showing prognostic relevance of LIG3 mRNA (NM_013975 transcript variant alpha) on OS and EFS of MM patients. **b** Analysis of GSE39683 dataset. *Left panel*: LIG3 mRNA levels in healthy donors and patients’ multiple myeloma and plasmacell leukemia. *Right panel*: DNA ligases mRNA levels in MM patients. *, *p* < 0.001; **, *p* < 0.0001. **c** Analysis of GSE9782 dataset. *Left panel*: LIG3 mRNA levels in MM patients with clinical response (R) or progressive disease (PD) after bortezomib treatment. *Right panel****:*** prognostic relevance of LIG3 mRNA levels on OS of MM patients enrolled to bortezomib-based therapy. *p < 0.05

To understand the role of DNA ligases in the pathophysiology of MM, the prognostic relevance of their expression was investigated by interrogating public MM datasets. Analysis of GSE24080 revealed that higher LIG3 mRNA expression significantly correlated with shorter overall survival (OS) and event-free survival (EFS), while LIG1 and LIG4 did not retain consistent prognostic relevance (Fig. 1a, Supplementary Fig.1a, b). Notably, LIG3 mRNA expression progressively increased in plasma cells from healthy donors (N) to MM and to plasma cell leukemia (PCL) patients and was the most expressed among DNA ligases (Fig.1b, Supplementary Fig.1c). Of note, no significant variation of copy number of LIG3 gene was found (Supplementary Fig. 1d), thus suggesting that high expression of LIG3 in MM patients could be derived from deregulation of transcriptional or post-transcriptional (e.g., miRNAs) mechanisms that normally regulate LIG3 expression. In addition, higher levels of LIG3 mRNA can be detected in high-risk MM group [39], in patients harboring deleterious cytogenetic abnormalities and in relapsed disease (Supplementary Fig. 1e, f). In particular, analysis of GSE9782 dataset revealed that higher levels of LIG3 mRNA significantly correlated with progressive disease (PD) and predicted poor OS for patient who received bortezomib-based therapy (Fig. 1c Supplementary Fig. 1g, h). Overall, these findings strongly suggest involvement of LIG3 in the MM genomic instability, which promotes disease progression and drug resistance.

### DNA ligase III as therapeutic target in MM

**Fig. 2:**
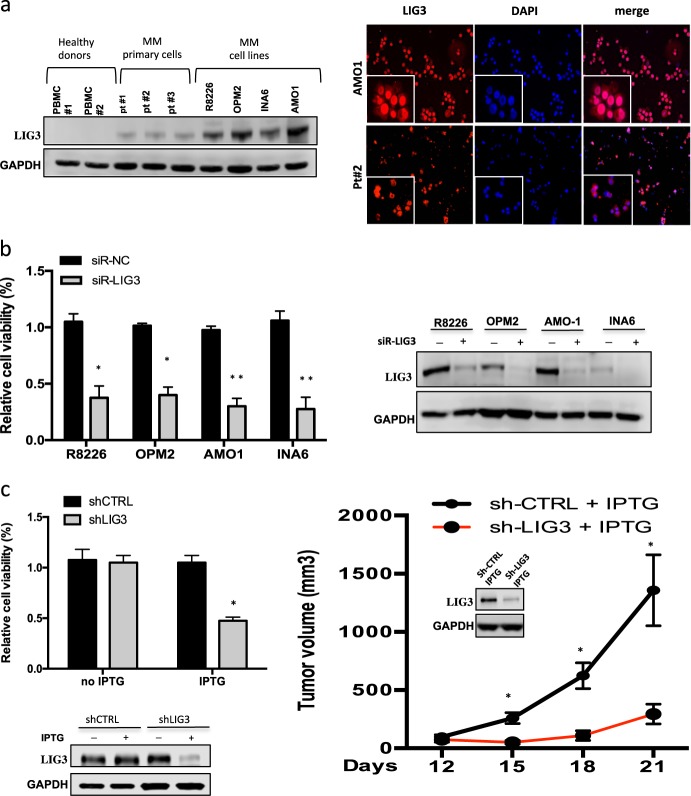
Addiction of MM cells to DNA ligase III. **a** *Left panel*: immunoblot of LIG3 performed on plasmacells from MM cell lines and patients (Pts) compared to peripheral blood mononuclear cells (PBMCs) collected from healthy donors. GAPDH was used as a loading control. *Right panel*: immunofluorescence analysis showing subcellular distribution of LIG3 in AMO1 and patient (Pt#2) MM cells. **b** *Left panel*: indicated MM cell lines were transfected with either scramble control or LIG3-siRNA clone #2. CTG assay was performed 96 h from transfection. Results are expressed as percentage of siR-NC-transfected cells. *Right panel:* LIG3 knockdown was confirmed by western blot analysis 48 h after transfection. **c** *Left panel*: AMO1 cells were transduced with IPTG inducible CTRL-shRNA or LIG3-shRNA lentiviral particles as described above. CTG assay was peformed 6 days from IPTG induction (Top). LIG3 knockdown was confirmed by Western blotting 4 days from IPTG induction (Bottom). *Rigth panel*: inducible IPTG CTRL-shRNA or LIG3-shRNA AMO1 cells were injected s.c. into SCID/NOD mice. Averaged tumor volume of each group ± SD and LIG3 protein knockdown from a representative AMO1 xenograft per group, are shown. *p < 0.05; **p < 0.01

Based on clinical findings, which suggested a potential important role of DNA ligase III in MM pathogenesis, its protein levels were analyzed in a panel of MM cell lines, primary cells from MM patients and healthy donors PBMCs. As shown in Fig. 2a, a significant upregulation of LIG3 protein expression in MM cells as compared to normal plasma cells, with a nuclear and cytoplasmic localization, was observed. To investigate its biological relevance in MM cells, knockdown experiments were performed to evaluate whether LIG3 was required for MM cell viability. Importantly, transient or stable LIG3 downregulation significantly reduced viability of MM cell lines (Fig.2b, Supplementary S2a).

To provide additional evidence of the relevance of LIG3-targeting, AMO1 cells were transduced with an inducible lentiviral vector by which LIG3 knockdown could be turned-on or -off upon IPTG addition or withdrawal. Inducible LIG3 downregulation resulted in a significant reduction of cell viability and clonogenicity in vitro and inhibition of tumor growth in vivo (Fig. 2c, Supplementary Fig. 2b,c).

Growth-inhibitory effects induced by LIG3 knockdown were associated to increase of unrepaired DNA double-strand breaks (DSBs), which finally led to apoptotic cell death (Supplementary Fig. 2d-f). Moreover, higher LIG3 protein levels were detected in AMO1 bortezomib-resistant cells (ABZB), as compared to their isogenic sensitive counterparts AMO1. Indeed, enforced expression of LIG3 on AMO1 cells significantly antagonized the effect induced by bortezomib on cell viability, according to what was observed by dataset analysis (Supplementary Fig. 2g); on the other hand, ABZB cells were extremely sensitive to LIG3-inhibition, as resulted by high impairment of survival (Supplementary Fig. 2h) observed after LIG3 knockdown.

Overall, these findings greatly suggest DNA ligase III as promising therapeutic target in MM.

### miR-22 is a negative regulator of LIG3 in MM

**Fig. 3:**
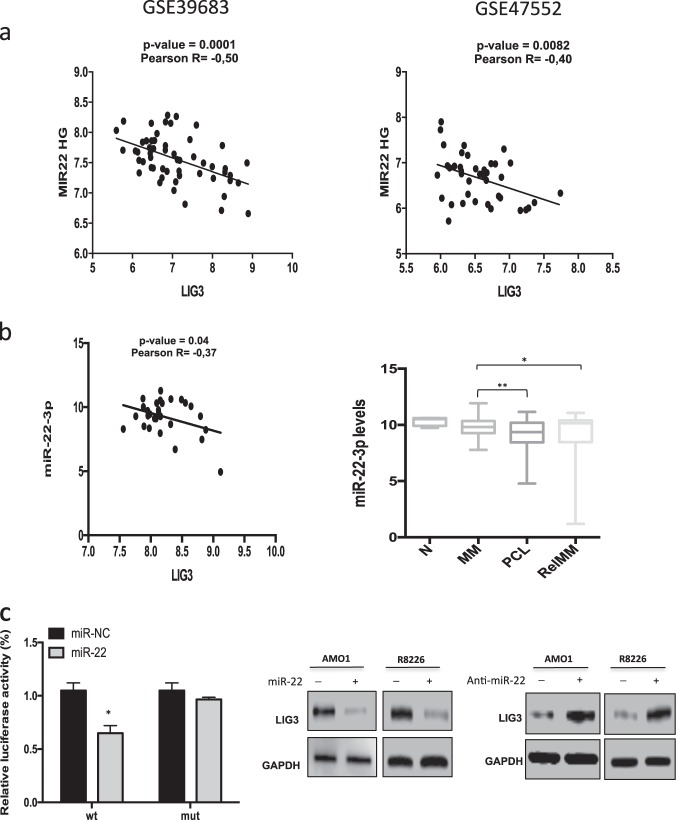
miR-22 as negative regulator of LIG3 expression in multiple myeloma patients. **a** Graphs of correlations between endogenous mRNA expression levels of LIG3 (NM_013975, transcript variant alpha) and MIR22HG in patient’s multiple myeloma cells from published datasets GSE39683 and GSE47552. **b** *Left panel*: graphs of correlations between endogenous mRNA expression levels of LIG3 and miR-22 in patient’s PCL cases from proprietary dataset. *Right panel:* miR-22-3p basal expression as evaluated by miRNA profiling on proprietary datasets. *p < 0.05, **p < 0.01. **c** *Left panel*: dual-luciferase assay performed on AMO1 cells co-transfected with firefly luciferase constructs containing the 3′ UTR of LIG3 or a deletion mutant (3′ UTR del) and miR-22 mimics or miR-NC as indicated. The data are shown as relative luciferase activity of miR-22–transfected cells compared with the control (miR-NC). Values represent mean ± SD of three different experiments. *p < 0.05. *Right panel***:** ectopic expression of miR-22 mimics or inhibitor in AMO1 and R8226 cells modulates protein levels of LIG3 as evaluated by immunoblot using GAPDH as loading control

To identify putative LIG3-targeting miRNAs, the microRNA Data Integration Portal was interrogated [40]. Five miRNAs, including miR-204-5p, miR-31-5p, miR-22-3p, miR-211-5p, and miR-146b-5p were predicted to target LIG3 with very high score range in more than 12 different data sources (Supplementary Fig. 3a). By an integrated approach [23] and the use of GSE39683 and GSE47552 public available datasets, a significant inverse correlation with LIG3 mRNA expression was found only for miR-22HG in both datasets, suggesting a potential activity of miR-22 as negative regulator of LIG3 in primary samples from MM patients (Fig. 3a). Consistent with these preliminary in silico findings, miRNA profiling of a series of patient-derived specimens (*n* = 4 normal, *n* = 97 MM, *n* = 30 PCL cases) confirmed a significant inverse correlation between miR-22 and LIG3 expression in PCL cases and downregulation of miR-22 during disease progression and in relapsed patients (Fig. 3b, Supplementary 3b). Then, miR-22 expression in a panel of MM cell lines, primary MM cells from patients and PBMC from healthy donors were analyzed by qRT-PCR. Importantly, a steady and overall downregulation of miR-22 in tumor cells as compared to normal cells was found (Supplementary Fig. 3c), opposite to the above described expression trend of LIG3 expression. Interestingly, the analysis of miR-22 levels in MM patients stratified according TC classification [41] showed that the TC2 subgroup, which is associated to hyperdiploid (HD) status, had the lower levels of miR-22, as compared to other subgroups. Since prior studies reported that HD MM patients showed high frequency of MYC rearrengements [42], a negative regulation of c-MYC on miR-22 transcription could be hypothesized. Indeed, siRNA-mediated knockdown of c-MYC induced a significant upregulation of miR-22 levels in AMO-1 MM cells, thus providing a possible explanation of miR-22 downregulation in these subgroups of MM patients (Supplementary Fig. 3d).

To provide a formal proof that LIG3 mRNA is targeted by miR-22, a luciferase reporter assay was performed. Luciferase activity was significantly reduced by miR-22 mimics in cells transfected with wild-type LIG3-reporter, while deletion of the miR-22 binding site (bases from 330 to 400 in the LIG3 3′-UTR, identified using microRNA.org) in the seed region of the LIG3 3′-UTR abrogated the miR-22 suppression of luciferase activity (Fig. 3c, Supplementary Fig.3d). Consistently with these results, transfection of miR-22 mimics or miR-22 inhibitor reduced or increased endogenous LIG3 protein levels (Fig. 3c, Supplementary Fig. 3e) in AMO1 and RPMI-8226 cells, respectively, indicating that miR-22 directly downregulates LIG3 expression.

### Enforced expression of miR-22 impairs growth and survival of MM cells, via targeting LIG3

After identification and validation of miR-22 as negative regulator of LIG3 expression, the effects of miR-22 overexpression on MM cells viability were evaluated. To this aim, AMO1, RPMI-8226, INA-6, and OPM2 MM cell lines were transduced with lentiviral vectors carrying miR-22 expression constructs. Importantly, stable overexpression of miR-22 strongly impaired MM cell viability (Supplementary Fig. 4a). To better evaluate these findings, AMO1 cells were transduced with a lentiviral construct by which miR-22 expression could be upregulated using a Tet-On system. Inducible overexpression of miR-22 by doxycycline produced knockdown of LIG3 protein and resulted in reduction of cell viability and clonogenic growth and induction of apoptosis (Supplementary Fig. 4b-d).

**Fig. 4:**
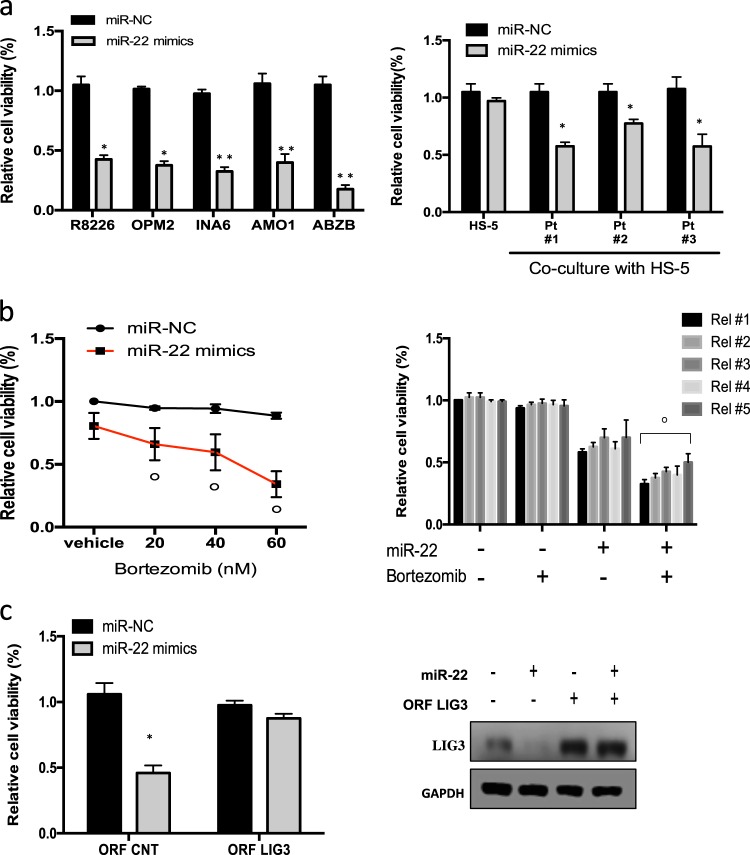
In vitro tumor suppressor activity of miR-22 in MM cells. **a** *Left panel***:** CTG assay was performed 96 h after transfection of indicated MM cell lines with miR-22 mimics or scrambled controls (miR-NC). *p < 0.01. *Right panel*: cell viability of CD138+ cells from three different MM patients co-cultured with HS-5 stromal cells and transfected with miR-22 mimics or miR-NC. The assay was performed 48 h after cell transfection. *p < 0.05, **p < 0.01. **b** CTG assay performed in ABZB cells (*left panel*) and in primary PCs from *n* = 5 relapsed MM patients (*right panel*) 48 h after co-treatment with miR-22 and bortezomib. *p < 0.05; °synergistic index > 1.0. **c** AMO1 cells were co-transfected with LIG3 ORF or control ORF and miR-22 mimics or miR-NC: l*eft panel*: CTG survival assay were performed 96 h after transfection. *p < 0.01. *Right panel:* immunoblot shows the levels of LIG3 and GAPDH 48 h after cell transfection

Then, the activity of miR-22 mimics on MM cells viability was investigated. Ectopic expression of miR-22 mimics induced significant reduction of cell viability and activation of apoptosis in MM cell lines (Fig. 4a, Supplementary Fig. 4e). Conversely, ectopic expression of miR-22 inhibitors did not significantly affect the viability of MM cells (Supplementary Fig. 4f). Importantly, transfection of miR-22 mimics impaired the viability of primary MM plasma cells (Fig. 4a) or INA6 cell line (Supplementary Fig. 4g) co-cultured with HS-5 stromal cells. This finding appears of relevance taking into account the pro-survival role of the MM-microenvironment. Moreover, miR-22 overexpression synergistically enhanced the sensitivity to bortezomib in resistant Abzb cells and in primary MM cells relapsed after bortezomib (Fig. 4b). Finally, to assess if the effect of miR-22 on survival and drug sensitization of MM cells was actually mediated via LIG3, AMO1 and ABZB cells were co-transfected with miR-22 and a miR-22-insensitive LIG3 gene expression construct lacking 3′UTR. Our results showed that the non-targetable LIG3 rescued transfected cells respectively from viability reduction and bortezomib-sensitization induced by miR-22 mimics (Fig. 4c, Supplementary Fig.4h).

### miR-22 inhibits LIG3-dependent DNA repair inducing DNA damage and apoptosis in MM cells

Since DNA ligase III plays a critical role in nuclear and mitochondrial DNA (mtDNA) repair, the effect of miR-22 on LIG3-dependent DNA repair of DSBs, the most dangerous DNA lesions, was investigated.

**Fig. 5:**
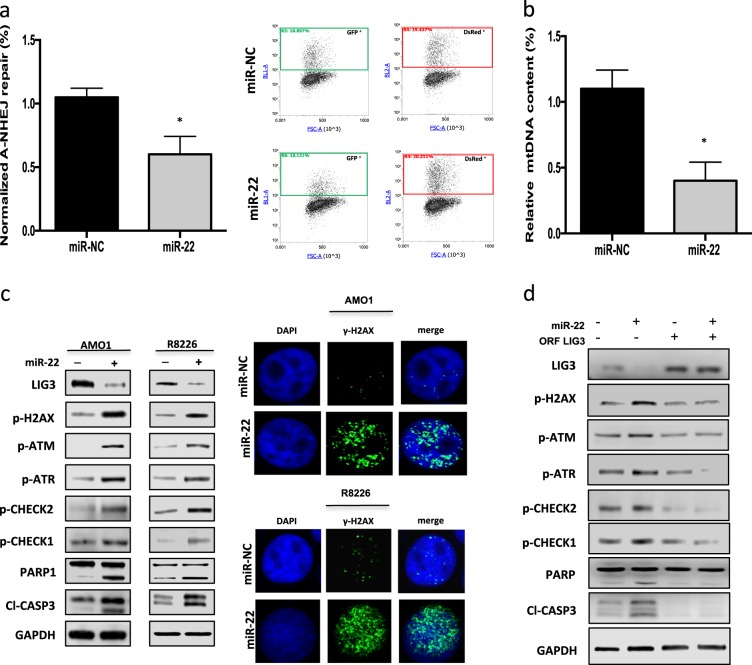
miR-22 overexpression counteracts LIG3 activity and induces DNA damage in MM cells. **a** Alt-NHEJ repair was evaluated by EJ2-GFP assay on AMO1 cells 48 h after transfection with miR-22 mimics or miR-NC. **b** Mitochondrial DNA copy number in AMO1 48 h after transfection. **c** AMO1 and R8226 cells were transfected with miR-22 mimics or miR-NC. *Left panel*: immunoblot analysis was performed 48 h after cell transfection. *Right panel:* γ-H2AX foci evaluation by immunofluorescence 48 h after cell transfection. Representative images of unrepaired DSBs are shown. DAPI (blue) was used for nuclear staining. **d** AMO1 cells were co-transfected with LIG3 ORF or control ORF and miR-22 mimics or miR-NC: immunoblot analysis was performed 48 h after cell transfection. *p < 0.05, **p < 0.01

First, the activity of Alt-NHEJ repair was evaluated by EJ2-GFP assay, a plasmid-based reporter assay specifically designed so the reconstitution of GFP predominantly reflect Alt-NHEJ event. Importantly, Alt-NHEJ repair was lower in MM cells transfected with miR-22 as compared to miR-NC (Fig. 5a). Consistently with reduction of this error-prone DNA repair pathway activity, SNP-array analysis confirmed an overall reduction of acquisition of new copy-number variations, including some high-risk chromosomal abnormalities [43] such as deletion of chromosome 1p22.2 and 14q, in miR-22-overexpressing cells (Supplementary Fig. 5a, b).

Then, influence on mtDNA metabolism was investigated. Transfection of miR-22 mimics induced a significant reduction of mtDNA content in MM cells (Fig. 5b) probably due to increase of unrepaired mtDNA damage. Consistently with mtDNA functions impairment increase of reactive oxygen species production (Supplementary Fig. 5c) was also observed after miR-22 mimics transfection. As consequence of LIG3-dependent DNA repair inhibition, enforced expression of miR-22 mimics induced a relevant increase in DNA DSBs along with a significant activation of DDR and apoptosis signaling, as evaluated by increased phosphorylation of ATM, ATR, CHECK1, CHECK2, H2AX, and cleavage of PARP1 and caspase-3 (Fig. 5c). Similar molecular perturbations were observed after miR-22 overexpression in primary CD138 + MM cells (Supplementary Fig. 5d). Cell cycle analysis revealed also G2-arrest after miR-22 transfection, which was abrogated by ATM/ATR inhibitor caffeine (Supplementary Fig. 5e). Moreover, co-treatment with caspase inhibitor Z-VAD-FMK rescued from apoptosis, but not from p-H2AX increase induced by miR-22 overexpression (Supplementary Fig.5f), thus suggesting that miR-22 tumor suppressor activity on MM cells relies on activation of DDR, which in turn leads to caspase-dependent cell death due to accumulation of irreparable DNA damage. Importantly, co-transfection of miR-22 with miRNA non-targetable LIG3-construct rescued AMO1 cells from DDR and apoptosis signaling activation, further confirming that miR-22-mediated DNA repair impairment and consequent DDR induction are primarily a result of LIG3 downregulation (Fig. 5d, Supplementary Fig. 5g, h).

### Inhibition of LIG3 by miR-22 induces anti-MM activity in vivo

**Fig. 6:**
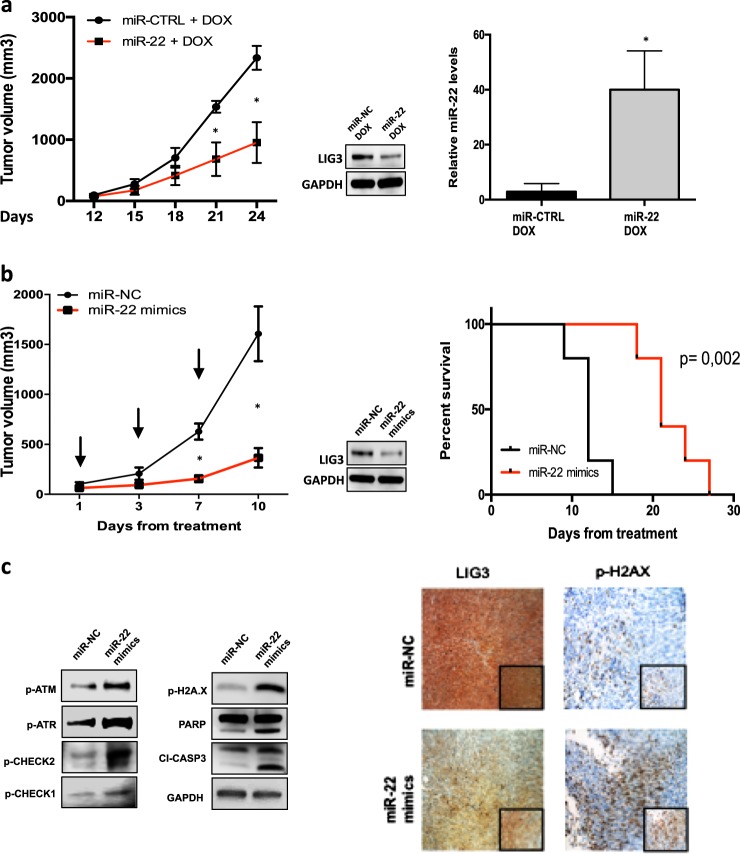
In vivo activity of miR-22 overexpression in MM xenograft models. **a** Inducible-CTRL or inducible-miR-22 cells were injected s.c. into SCID/NOD mice. When palpable tumors became detectable, animals were randomized to receive either vehicle (5% sucrose) or doxycycline (2 mg/mL in 5% sucrose) via drinking water for duration of study. *Left panel*: averaged tumor volume of each group ± SD is shown; *p < 0.01. *Right panel*: LIG3 knockdown and miR-22 overexpression were confirmed respectively by western blot analysis and qRT-PCR from a representative AMO1 xenograft per group. *p < 0.01. **b** In vivo growth of luciferase gene-marked AMO1 xenografts intra-tumorally treated with miR-22 mimic or scramble controls. Palpable subcutaneous tumor xenografts were treated with 20 mg of NLE-formulated oligos. Intra-tumor injections were administered every other day, for a total of three injections (indicated by arrows). *Left panel:* averaged tumor volume of each group ± SD is shown. *p < 0.01. *Right panel:* survival curves (Kaplan–Meier) of each group (log-rank test, *p* < 0.05). Survival was evaluated from the first day of treatment until death or sacrifice. Percentage of mice alive is shown. **c** *Left panel*: immunoblot of p-ATM, p-ATR, p-CHECK1, p-CHECK2, cleaved-caspase-3, and PARP in lysates from a representative AMO1 xenograft per group. GAPDH was used as a loading control. *Right panel*: IHC analysis (20×, 40x insets) from a representative AMO1 xenograft per group for LIG3 and p-H2AX expression

To investigate the translational relevance of our in vitro findings, subcutaneous MM xenografts in NOD-SCID mice using the inducible AMO1-ctrl and AMO1-miR-22 cells were generated. Doxycycline treatment was started after the tumor was established. Animals bearing AMO-miR-22 xenografts showed a significant inhibition of tumor growth as compared to AMO-ctrl tumors (Fig. 6a).

Next, in vivo anti-MM activity of formulated miR-22 mimics was evaluated in NOD-SCID mice bearing subcutaneous AMO1 xenografts. Luciferase gene-marked AMO1 xenografts were intra-tumorally treated every other day for a total of three injections, with 1 mg/kg of oligos formulated with NLE particles, a strategy specifically designed for delivery of oligonucleotides in vivo. miR-22 mimics treatment resulted in a significant tumor-growth inhibition and prolonged survival (Fig. 6b, Supplementary Fig. 6a,b). Consistent with in vitro data, downregulation of LIG3 protein and increased expression of DDR, in tumors retrieved from miR-22 mimics treated animals (Fig. 6c), were found.

## Discussion

DNA ligases are essential enzymes for DNA repair and replication, since they catalyze the last step in which DNA breaks are joined. In this study, we investigated the potential involvement of DNA ligases in the genomic instability and survival of MM cells. By comparing prognostic relevance of DNA ligases mRNA expression through whole gene expression data set analyses, we found that high LIG3 mRNA levels were significantly correlated to worse outcome in MM patients and increased during progression of disease and in relapsed patients. These findings, were consistent with the pivotal role played by DNA ligase III in Alt-NHEJ, an highly error-prone DNA repair pathway, which is strongly involved in the genomic instability, chromosome translocations, and drug resistance of different tumors, such as leukemia, lymphoma, neuroblastoma, and breast cancer [44–47]. In addition to its role on nuclear DNA repair, LIG3 gene encodes also the principal DNA ligase involved in mtDNA replication and repair, essential requisite to sustain mitochondrial contribute to cancer cell survival [48–50]. Taking into account that these factors are of major relevance in MM pathobiology [51, 52], at our knowledge we show for the first time, that LIG3 knockdown strongly increases DNA damage and finally inhibits MM cell growth in vitro and in vivo. Our findings therefore offer proof of concept for a new DNA repair addiction in MM. We attempted to investigate mechanisms leading to LIG3 upregulation and we focused on miRNAs since their important regulatory role of DDR machinery, still unexplored in MM. To this aim, we performed an integrated bioinformatics analysis of miRNAs and mRNAs expression profiles, which showed that miR-22 inversely correlated with LIG3 mRNA, indicating the relevance of miR-22 as LIG3 negative regulator in MM patients. On this basis, we confirmed and validated for the first time LIG3 mRNA as direct miR-22 target. Consistently, miR-22 was found downregulated in patients with more advanced stage, as well as in MM cell lines, in contrast to what observed for LIG3 expression. Altogether, these findings suggested a potential involvement of miR-22 in the regulation of LIG3-driven DNA repair and prompted us to investigate the role of this miRNA in MM by orthogonal approach.

The human miR-22 gene is located on chromosome 17 (17p13.3), a frequently hypermethylated, deleted or loss of heterozygosity-associated region in cancer, including MM. Indeed, miR-22 expression was significantly affected by chromosome arm 17p loss in a representative panel of primary MM tumors [53]. According to previous studies in other malignancies [54], our findings suggest that miR-22 downregulation in MM could be related to transcriptional repression exerted by c-MYC, a known master regulator of MM pathogenesis.

MiR-22 plays a crucial role in a variety of cellular processes and its role in cancer diverges in different malignancies, being able to act both as tumor suppressor and oncogenic miRNA depending on the molecular contexts [55, 56].

We here provide evidence that miR-22 acts as tumor suppressor in MM cells in vitro and in vivo, via LIG3 targeting. Consistently, ectopic expression of miR-22 significantly inhibits LIG3-mediated nuclear and mtDNA repair, strongly increasing unrepaired DNA damage that finally led to apoptotic cell death of MM cells. Importantly, our data show that the inhibition of Alt-NHEJ repair by miR-22 ectopic expression significantly reduces the acquisition of new genetic changes in MM cells, thus suggesting a protective role of miR-22 against genomic instability, which foster disease progression and drug resistance. Interestingly, we found that upregulation of LIG3 is associated to bortezomib resistance and that LIG3 downregulation is highly toxic and partially restore drug sensitivity in bortezomib-resistant cells. These findings could be consistent with recent evidence which showed that bortezomib resistance in malignant plasmacells is associated to high dependency on increased mitochondrial activity and that mitochondrial activity modulation decreases bortezomib resistance [57–59].

**Fig. 7:**
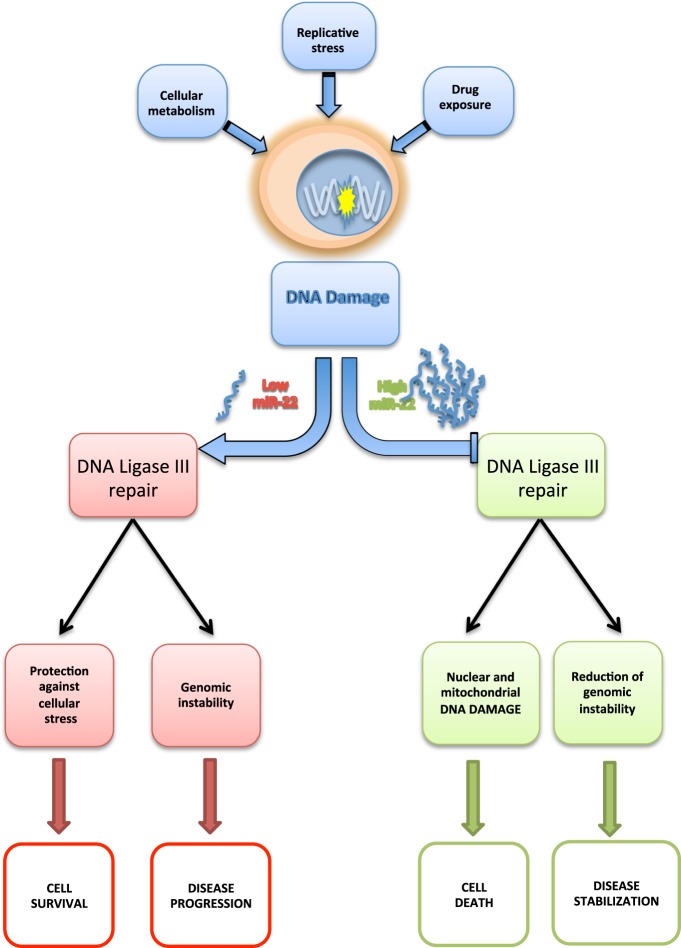
Esplicative cartoon of miR-22-mediated modulation of LIG3-driven DNA repair

On these findings, we postulated that MM cells, in order to survive to ongoing endogenous (oxidative and replicative stress) or drug mediated (e.g., bortezomib) DNA damage, switch DNA repair machinery to LIG3-driven-DNA repair, which is able at the same time, to repair nuclear and mtDNA and to allow the acquisition of new genetic changes, leading to disease progression and drug resistance (Fig. 7). In this light, our findings demonstrate that MM cells are addicted to DNA ligase III. Moreover, the inhibitory activity exerted by miR-22 on LIG3 demonstrates a novel axis of genome stability regulation and survival in MM.

## Electronic supplementary material

Supplementary Material and methods

Supplementary Figures

Legends to Supplementary Figures
