# Supplementary material for: miR-22 suppresses DNA ligase III addiction in multiple myeloma
Source: Leukemia. 2018 Aug 17;33(2):487–98. doi: 10.1038/s41375-018-0238-2 (PMC6365379; doi:10.1038/s41375-018-0238-2)
Supplement: Supplementary file 1 — Supplementary Material and methods [file 41375_2018_238_MOESM1_ESM.pdf]

Supplementary Material and Methods

Multiple myeloma cell lines, primary cells, and reagents

Peripheral blood mononuclear cells (PBMCs) and primary cells from multiple myeloma patient bone marrow aspirates, following informed consent and University Magna Graecia (Catanzaro, Italy) IRB approval, were isolated using Ficoll–Hypaque density gradient sedimentation as reported previously (1). Multiple myeloma patients' cells were separated from bone marrow samples by antibody-mediated selection using anti-CD138 magnetic-activated cell separation microbeads (Miltenyi Biotec). Purity of immunoselected cells was assessed by flow-cytometry analysis using a phycoerythrin-conjugated CD138 monoclonal antibody by standard procedures. CD138+ cells from MM patients pt#1, pt#2 and pt#3 were cultured in RPMI-1640 medium (Gibco®, Life Technologies) supplemented with 20% fetal bovine serum (Lonza Group Ltd.) and 1% penicillin/streptomycin (Gibco®, Life Technologies). Multiple myeloma cell lines (HMCLs) AMO1 were purchased from DSMZ (Braunschweig, Germany). RPMI-8226 and OPM2 were purchased from ATCC (Manassas, VA, USA). AMO1 bortezomib-resistant (ABZB) were kindly provided by Dr. Christoph Driessen (Eberhand Karls University, Tübingen, Germany). Multiple myeloma cell lines were cultured in RPMI1640 (Gibco, Life Technologies) supplemented with 10% FBS (Lonza Group). INA-6 cell line (kindly provided by Dr. Renate Burger, University of Erlangen- Nuernberg, Germany) was cultured in the presence of recombinant human IL6 (2,5 ng/mL, R&D Systems, Minneapolis, MN); this cell line was not further authenticated but confirmed for the described IL6 dependence. HS-5 human stromal cell line (purchased from ATCC, CRL-11882TM) was cultured in DMEM supplemented with 10% fetal bovine serum and 1% penicillin/streptomycin . Co-culture experiments were performed in 6 well plate at a density of 2,5 × 10 5 cells/ ml in 1:1 HS-5 /MM cells ratio. All these cell lines were immediately frozen and used from the original stock within 6 months.

Virus Generation and Infection of cells.

The human LIG3 was knocked down using the pLKO.1-puro vector as well as pLK-puro-IPTG-3XLacO vector containing the target sequence (indicated in the list below) or scramble control, according to the manufacturer's specifications (mission shRNA set, Sigma Aldrich). To obtain stable silenced cells, lentiviral particles were produced and transduced as previously described (1) and the knockdown efficiency was validated by detecting LIG3 protein level by western blot analysis and RT-qPCR analysis. For the inducible model, MM cells expressing pLK-puro-IPTG-3XLacO shRNA were treated with or without IPTG (1mM) daily to obtain LIG3 knockdown. The efficacy of the induction was confirmed by western blot and RT-qPCR analysis.

MM cells stably expressing miR-22 gene were transduced with Lenti-miR-22 microRNA precursor constructs (System Biosciences, CA, US); lentiviral particles were produced and transduced as previously described. For inducible miRNA overexpression, AMO1 cells were transduced with shMIMIC Inducible Lentiviral microRNA or specific negative control (GE Dharmacon, Pittsburgh, PA) and then treated with or without doxycycline (500 ng/mL) every other day. miR-22 overexpression efficiency was evaluated by RT-qPCR.

To generate cells stably expressing luciferase transgene, AMO-1 cells were transduced with pLenti-III- PGK-Luc (ABM Inc.) vector.

RNA extraction and quantitative real-time-PCR.

Total RNA extraction from MM cells and qRT-PCR were performed as previously described (2). Briefly, total RNA was extracted from cells using TRIzol® reagent (Gibco, Life Technologies, Carlsbad, CA), following the manufacturer's instructions. The RNA quantity and quality was assessed through NanoDrop® (ND-1000 Spectrophotometer). To evaluate gene expression levels, 1000 ng of total RNA were reverse transcribed to cDNA using the "High Capacity cDNA Reverse Transcription Kit" (Applied Biosystems, Carlsbad, CA). The single-tube TaqMan assay (Applied Biosystems, Carlsbad, CA) was used to detect and quantify LIG3 (Hs00242692_m1), according to the manufacturer's instructions, using Viia 7 Dx multicolor detection system (Applied Biosystems, Carlsbad, CA). The obtained Threshold Cycle (CT) values were normalized on GAPDH (Hs03929097_g1). Comparative real-time polymerase chain-reaction (RT- PCR) was carried out in triplicate, including no-template controls. Relative expression was calculated using the comparative cross threshold (Ct) method. Taq-Man® MicroRNA assays (Life Technologies) was used to detect and quantify mature mir-22-3p (assay ID 000398), according to manufacturer's guidelines on a ViiA7 System (Life Technologies). MiR-22-3p expression was normalized on RNU44 (assay ID 001094).

Gene expression profiling

Highly purified PC samples (CD138 ≥ 90%) from the bone marrow of 129 multiple myeloma (MM), 36 plasma cell leukemia (PCL) and 20 relapsed patients together with 4 healthy donors (N) have been profiled on the GeneChip® Human Gene 1.0 ST array (Affymetrix, Santa Clara, CA) (3). A subset of them (97 MM, 30 PCL, 13 relapsed cases and 4 N) have also been profiled on GeneChip® miRNA 3.0 arrays (Affymetrix, Santa Clara, CA). Gene and miRNA expression profiling data were generated as previously described(4), using Brainarray annotation procedure (http://brainarray.mbni.med.umich.edu/Brainarray/Database/CustomCDF/18.0.0/version.html). The GEP and miRNA expression data have been deposited in the NCBI Gene Expression Omnibus database (GEO; http//www.ncbi.nlm.nih.gov/geo; accession No. GSE66293, GSE73452 and GSE70254).

In vitro transfection of MM cells

Synthetic *mir*Vana™ miR-22-3p mimic and inhibitor were purchased from Invitrogen™ (Thermo Scientific). Stealth RNAi™ LIG3 siRNAs (clone IDs: #1-HSS106054, #2-HSS106055, #3-HSS180680) and Silencer™ Select MYC siRNA (clone IDs: s9129) were purchased from Invitrogen™ (Thermo Scientific). All the oligos were used at 100 nmol/L final concentration. MM cells were transfected using Neon Transfection System (Invitrogen™) (2 pulse at 1.050 V, 30 milliseconds). The same conditions were applied for transfection of MM cells with 2,5 μg of expression vectors carrying the ORFs of LIG3 NM_013975 (EX-E2138-M68), with empty vector (CNT) used as control (EX-NEG-M68) (GeneCopeia, Rockville, MD, USA).

Cell cycle analysis

Analysis of cell cycle was performed by Propidium Iodide flow cytometry assay (BD Pharmingen), according to manufacturer's instructions. Flow cytometry analysis was performed by Attune NxT Flow cytometer (Thermo Fisher Scientific).

DSB repair assay

In vivo DSB repair assays were performed as previously described (5, 6). Briefly, EJ2-GFP plasmid (#44025, Addgene) was linearized with I-SceI (Thermo Scientific) digestion and transfected into 1x10^6 cells at a ratio of 1ug per well. In parallel, cells were transfected using with 0.1 µg of DsRed-N1 plasmid (kindly provided by Dr.Michele Cea, Dana-Farber Cancer Institute, Boston, MA) as the internal control. 48h after miR-NC or miR-22 transfection, the numbers of GFP+ and DsRed+ cells were determined by flow cytometry (Attune NxT, Thermo Fisher Scientific). For each experiment, FACS analyzed a minimum of 20,000 cells. The ratio between GFP+ and DsRed+ cells was used as a measure Alt-NHEJ repair efficiency.

Western blot analysis

Whole cell protein extracts were prepared from MM cells and from PBMCs in NP40 CellLysis Buffer (Novex®) containing a cocktail of protease inhibitors (Sigma, Steinheim, Germany). Cell lysates were loaded and PAGE separated. Proteins were transferred by Trans-Blot® TurboTM Transfer Starter System for 7 min. After protein transfer, the membranes were blotted with antibodies listed in the table and visualized with C-DiGit® Blot Scanner (LI-COR) by using the ECL Western Blotting Detection Reagents (Thermo Fisher Scientific, IL). Image capture was carried out using image studio® (LI-COR, version 5.0) software.

Luciferase reporter experiments

Renilla luciferase from pCMV-RL was included to normalize firefly luciferase activity. The 3' untranslated region (UTR) of LIG3 (NM_013975) was cloned in pEZX-MT01 vector and purchased from GeneCopoeia (Rockville, MD). Multiple myeloma cells were electroporated using 2.5 ug of the firefly luciferase reporter; for each plate, 100 nmol/L of the synthetic miR-22 or miR-NC and 0.5 ug of pCMV-RL were used. Firefly and Renilla luciferase activities were measured using the Dual-Luciferase Assay Kit (Promega). Data are expressed as the ratio of luminescence from firefly divided by luminescence from Renilla luciferase.

Mitochondrial DNA measurements

To assess mitochondrial DNA (mtDNA) copy number, genomic DNA was extracted from cells using the Perfect Pure DNA Blood kit (5 Prime). The relative mtDNA copy number was determined by a real-time polymerase chain reaction, according manifacturer's instructions (human real-time PCR mitochondrial DNA damage analysis kit, Detroit R&D).

ROS Assay

ROS levels were measured by ROS-Glo™ H2O2 assay (Promega), according to the manufacturer's instructions.

Immunofluorescence

Cells were harvested, centrifuged onto glass slides (Cytospin 4, Thermo Scientific), and fixed in 4% paraformaldehyde in PBS, pH 7.4, for 12 min at 22°C, followed by three 5-min washes in PBS. Cells were permeabilized (0.1% Triton X-100 in PBS, 15-min), washed in PBS (3×, 5 min each), and incubated 1 h at

22°C with blocking buffer (1.5% BSA in PBS). They were reacted >12 h at 4°C with primary antibodies listed in the table, washed in PBS (3×, 5 min each), and incubated 1 h at 22°C in the dark, with appropriate secondary antibodies. Cells were washed 3× in PBS and mounted under coverslips with Vectashield with DAPI (Vector Laboratories). Images were acquired with an SP2 Leica Zeiss confocal laser-scanning microscope with a 63× oil objective.

SNP-array data analysis

DNA was extracted from the AMO1 miR-NC and AMO1 miR-22 overexpressing cells using the Perfect Pure DNA Blood kit (5 Prime) and analyzed using the Affymetrix Cytoscan HD array (Affymetrix, Inc., Santa Clara, CA) according to manufacturers's instructions, to estimate genomic instability and ongoing DNA rearrangements. This array consists of 2.67 million markers for copy number variation (CNV) analysis, including 750,000 SNP and 1.9 million non-polymorphic probes, with an average spacing for RefSeq genes of 880 bp. Analysis of intensity data (CEL file) was performed with Chromosome Analysis Suite v 3.1 (ChAS 3.1) software using the Affymetrix HapMap Reference Model File for comparison. We used 25 probes and > 25 kb and >50 kb as a minimum cutoff for deletions and gains respectively. Map position was based on GRCh37/hg19 assembly. Genotype from cell samples harvested at the beginning of experiment (day 0), were used as baselines to identify new CNVs in the treated cells.

Animals and in vivo models of human MM

Male CB-17 severe combined immunodeficient (SCID) mice (6- to 8-weeks old; Harlan Laboratories, Inc., Indianapolis) were housed and monitored in our Animal Research Facility. Experimental procedures and protocols had been approved by the Magna Graecia University IRB and conducted according to protocols approved by the National Directorate of Veterinary Services (Italy). Mice were subcutaneous inoculated with 5x10^6 AMO1 cells and treatment started when palpable tumors became detectable (100–200 mm^3). Tumor sizes were measured as described (2), and the investigator was blinded to group allocation. Tumor size of luciferase gene-marked AMO1 xenografts was also measured by IVIS Lumina II. Oligos were NLE-formulated within MaxSuppressor In Vivo LANCEr II (BIIo Scientific) to achieve an efficient delivery, as reported (7, 8). ***In vivo* inducible model.** For the xenograft inducible mouse-model, we subcutaneously implanted 5 x 10^6 AMO1 cells with inducible expression scramble or shRNA targeting LIG3 in NOD-SCID mice (Charles River Laboratories). After tumors became palpable (100–200 mm^3), IPTG (Sigma, 10mM) was added to drinking water every other day for mice bearing tumors. In the second experiments, we subcutaneously implanted 5 x 10^6 AMO1 cells with inducible expression CTRL-tet-on or miR-22-tet-on AMO1 cells. After tumors became palpable (100–200 mm^3), doxycycline (Sigma) was added every other day to the water (2 mg/ml in 5% sucrose). Tumor sizes were measured as described (2) and the investigator was blinded to group allocation.

Histology and immunohistochemistry

Retrieved tumors from animals were fixed in 4% buffered formaldehyde and 24 hours later washed, dehydrated, and embedded in paraffin. For light microscopy analysis by an optical microscope Nikon i55 (Nikon Corporation, Tokyo, Japan), we performed staining with H&E on 4-mm tumor sections mounted on poly-lysine slides. For IHC staining, 2-mm–thick tumor slices were de-paraffinized and pretreated with the

Epitope Retrieval Solution 2 (EDTA buffer, pH 8.8) at 98 C for 20 minutes. After washing steps, peroxidase blocking was carried out for 10 minutes using the Bond Polymer. All procedures were performed using the Bond Max Automated Immunohistochemistry. Tissues were washed and incubated with the primary antibody directed against Ki-67 (Dako, clone MIB-1; 1:150), DNA Ligase III (Genetex, 1:500) and p-H2AX (Cell Signaling, 1:480). Subsequently, tissues were incubated with polymer for 10 minutes and developed with DAB–Chromogen for 10 minutes. Slides were counterstained with Hematoxylin.

List of shRNA vectors

**Clone IDs**	**Target sequence**
pLKO.1 LIG3 shRNA TRCN0000310601	CCGGGCCCACTTTAAGGACTACATTCTCGAGAATGTAGTCCTTAAAGTGGGCTTTTTG
pLKO.1 LIG3 shRNA TRCN0000300259	CCGGCCGGATCATGTTCTCAGAAATCTCGAGATTTCTGAGAACATGATCCGGTTTTTG
pLKO.1 LIG3 shRNA TRCN0000048499	CCGGCCGGATCATGTTCTCAGAAATCTCGAGATTTCTGAGAACATGATCCGGTTTTTG
pLKO-puro-IPTG-3XLacO LIG3 inducible shRNA TRCN0000310601	CCGGGCCCACTTTAAGGACTACATTCTCGAGAATGTAGTCCTTAAAGTGGGCTTTTTG

List of Antibodies

**Antibodies**	**Sources**	**Catalog #**	**Applications**
Cleaved-Caspase 3 (Asp175)	Cell Signaling Technology	9661	WB (1:1000)
GAPDH	Santa Cruz	25778	WB (1:1000)
PARP	Cell Signaling Technology	9532	WB (1:1000)

Phospho-CHK1 (Ser345)	Cell Signaling Technology	2348	WB (1:1000)
phospho-CHK2 (Thr 68)	Cell Signaling Technology	2197	WB (1:1000)
phospho-ATM (Ser 1981)	Cell Signaling Technology	5883	WB (1:1000)
phospho-ATR (Ser 428)	Cell Signaling Technology	2853	WB (1:1000)
phospho-Histone H2A.X (Ser139)	Cell Signaling Technology	9718	WB (1:1000)<br>IF (1:200)<br>IHC (1:480)
DNA Ligase III	Genetex	GTX103172	WB (1:1000)<br>IF (1:500)<br>IHC (1:500)
c-Myc	Cell Signaling Technology	5605	WB (1:1000)
Goat anti-rabbit IgG-HRP	Santa Cruz	2054	WB (1:3000)
Goat anti-mouse IgG-HRP	Santa Cruz	2055	WB (1:3000)

WB, western blot. ChIP, chromatin immunoprecipitaion. IF, immunofluorescence. IHC, immunohistochemistry

References

1. Amodio N, Di Martino MT, Foresta U, Leone E, Lionetti M, Leotta M, et al. miR-29b sensitizes multiple myeloma cells to bortezomib-induced apoptosis through the activation of a feedback loop with the transcription factor Sp1. Cell death & disease. 2012;3:e436.
2. Leone E, Morelli E, Di Martino MT, Amodio N, Foresta U, Gulla A, et al. Targeting miR-21 inhibits in vitro and in vivo multiple myeloma cell growth. Clinical cancer research : an official journal of the American Association for Cancer Research. 2013;19(8):2096-106.
3. Lionetti M, Barbieri M, Todoerti K, Agnelli L, Marzorati S, Fabris S, et al. Molecular spectrum of BRAF, NRAS and KRAS gene mutations in plasma cell dyscrasias: implication for MEK-ERK pathway activation. Oncotarget. 2015;6(27):24205-17.
4. Calura E, Bisognin A, Manzoni M, Todoerti K, Taiana E, Sales G, et al. Disentangling the microRNA regulatory milieu in multiple myeloma: integrative genomics analysis outlines mixed miRNA-TF circuits and pathway-derived networks modulated in t(4;14) patients. Oncotarget. 2016;7(3):2367-78.
5. Bennardo N, Cheng A, Huang N, Stark JM. Alternative-NHEJ is a mechanistically distinct pathway of mammalian chromosome break repair. PLoS genetics. 2008;4(6):e1000110.
6. Czochor JR, Sulkowski P, Glazer PM. miR-155 Overexpression Promotes Genomic Instability by Reducing High-fidelity Polymerase Delta Expression and Activating Error-Prone DSB Repair. Molecular cancer research : MCR. 2016;14(4):363-73.
7. Raimondi L, Amodio N, Di Martino MT, Altomare E, Leotta M, Caracciolo D, et al. Targeting of multiple myeloma-related angiogenesis by miR-199a-5p mimics: in vitro and in vivo anti-tumor activity. Oncotarget. 2014;5(10):3039-54.
8. Morelli E, Leone E, Cantafio ME, Di Martino MT, Amodio N, Biamonte L, et al. Selective targeting of IRF4 by synthetic microRNA-125b-5p mimics induces anti-multiple myeloma activity in vitro and in vivo. Leukemia. 2015;29(11):2173-83.
