# Supplementary material for: miR-22 suppresses DNA ligase III addiction in multiple myeloma
Source: Leukemia. 2018 Aug 17;33(2):487–98. doi: 10.1038/s41375-018-0238-2 (PMC6365379; doi:10.1038/s41375-018-0238-2)

Supplementary Fig.1

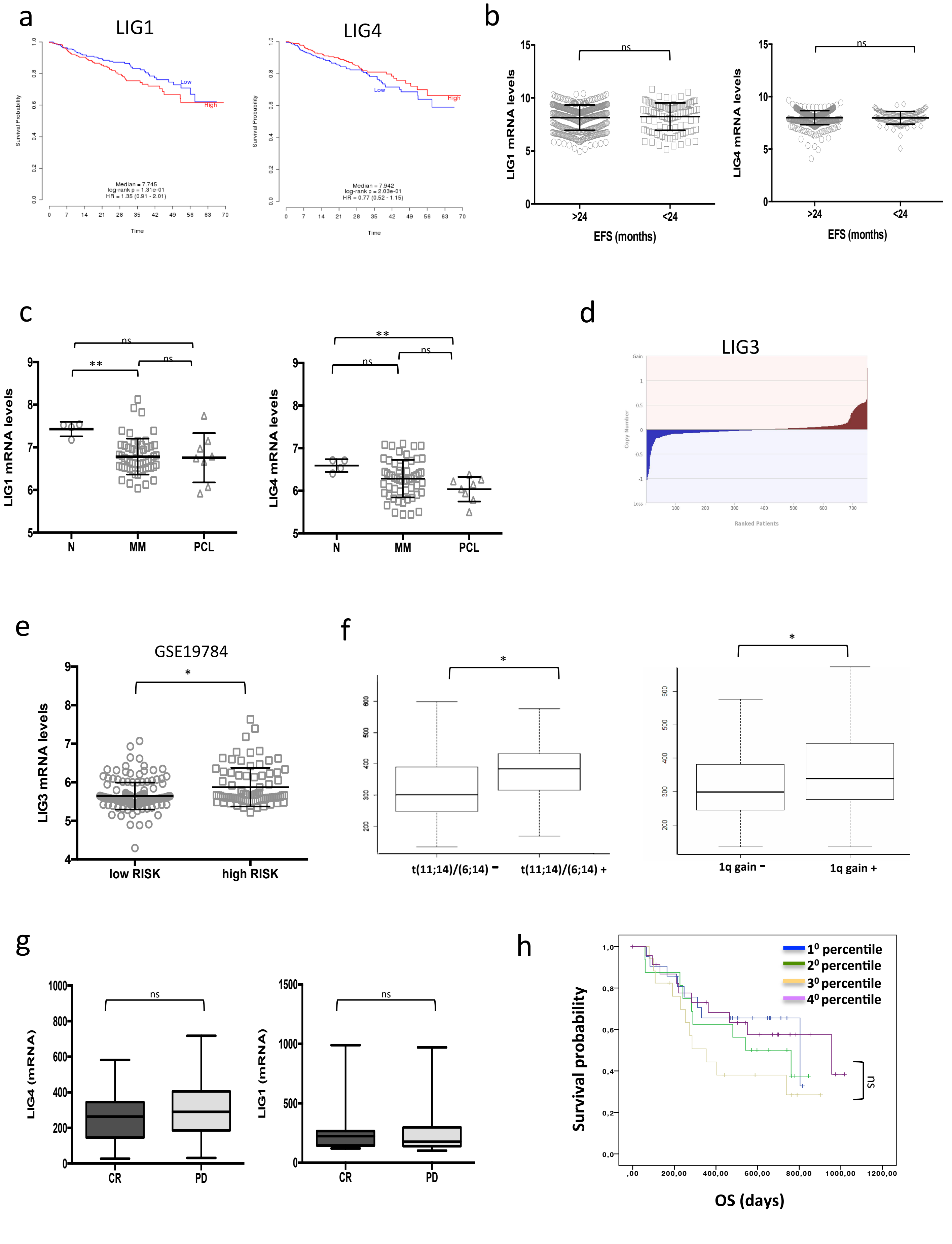

Supplementary Fig.2

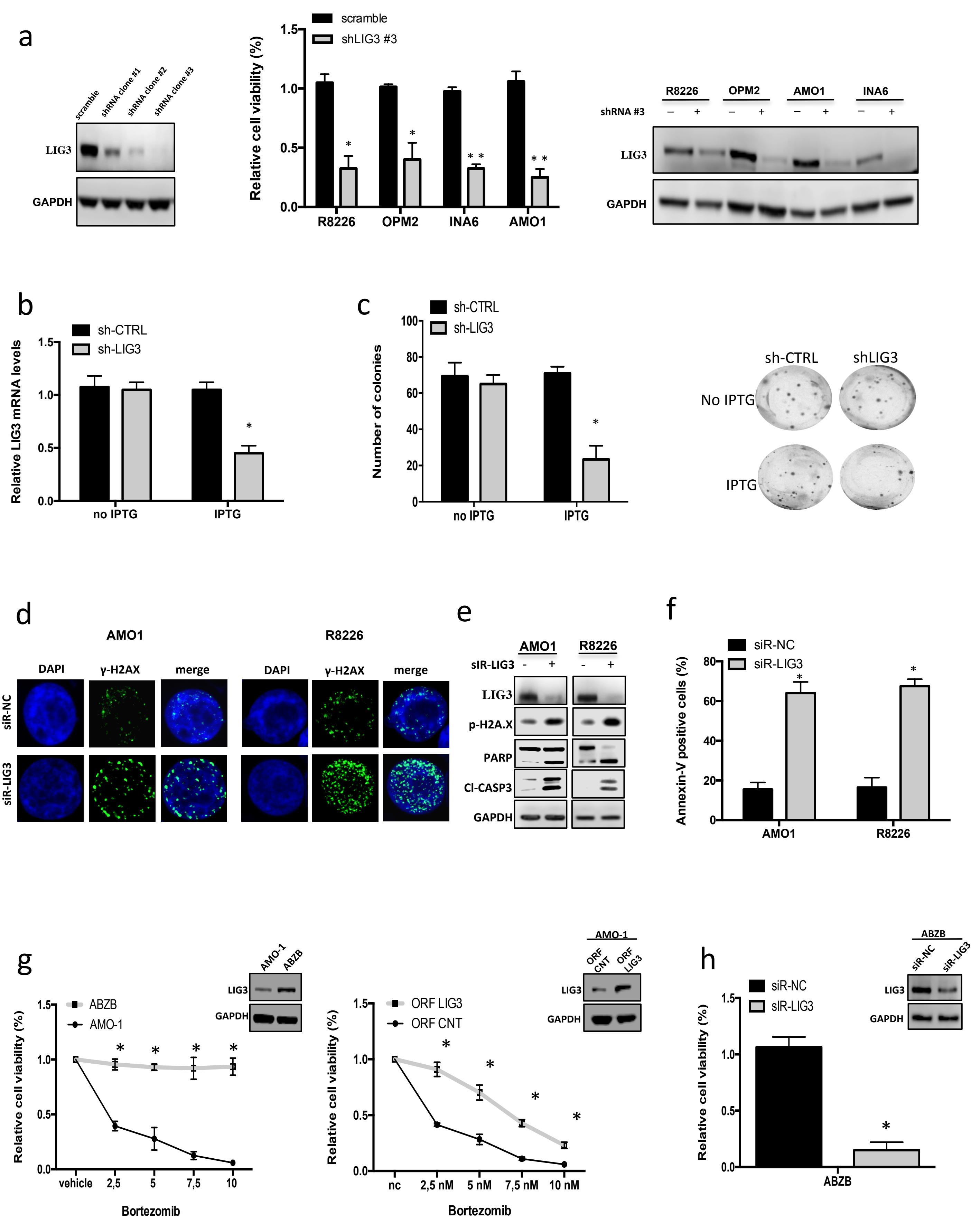

Supplementary Fig.3

a

GENE SYMBOL	microRNA	Integrated score	Number of source	Score Class
LIG3	hsa-miR-204-5p	0.466	13	Very high
LIG3	hsa-miR-211-5p	0.439	13	Very high
LIG3	hsa-miR-22-3p	0.430	12	Very high
LIG3	hsa-miR-31-5p	0.390	12	Very high
LIG3	hsa-miR-146b-5p	0.385	13	Very high

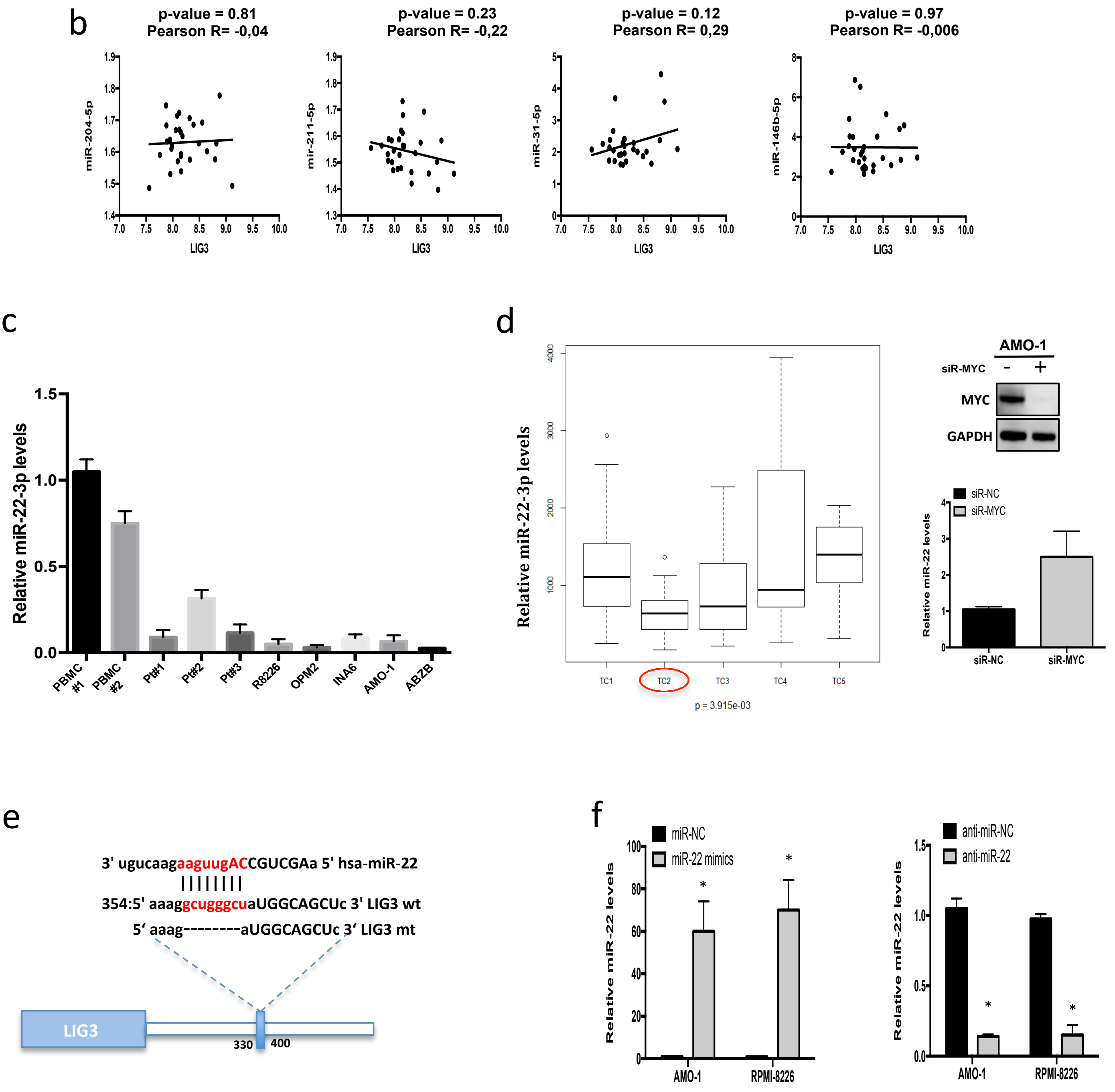

Supplementary Fig.4

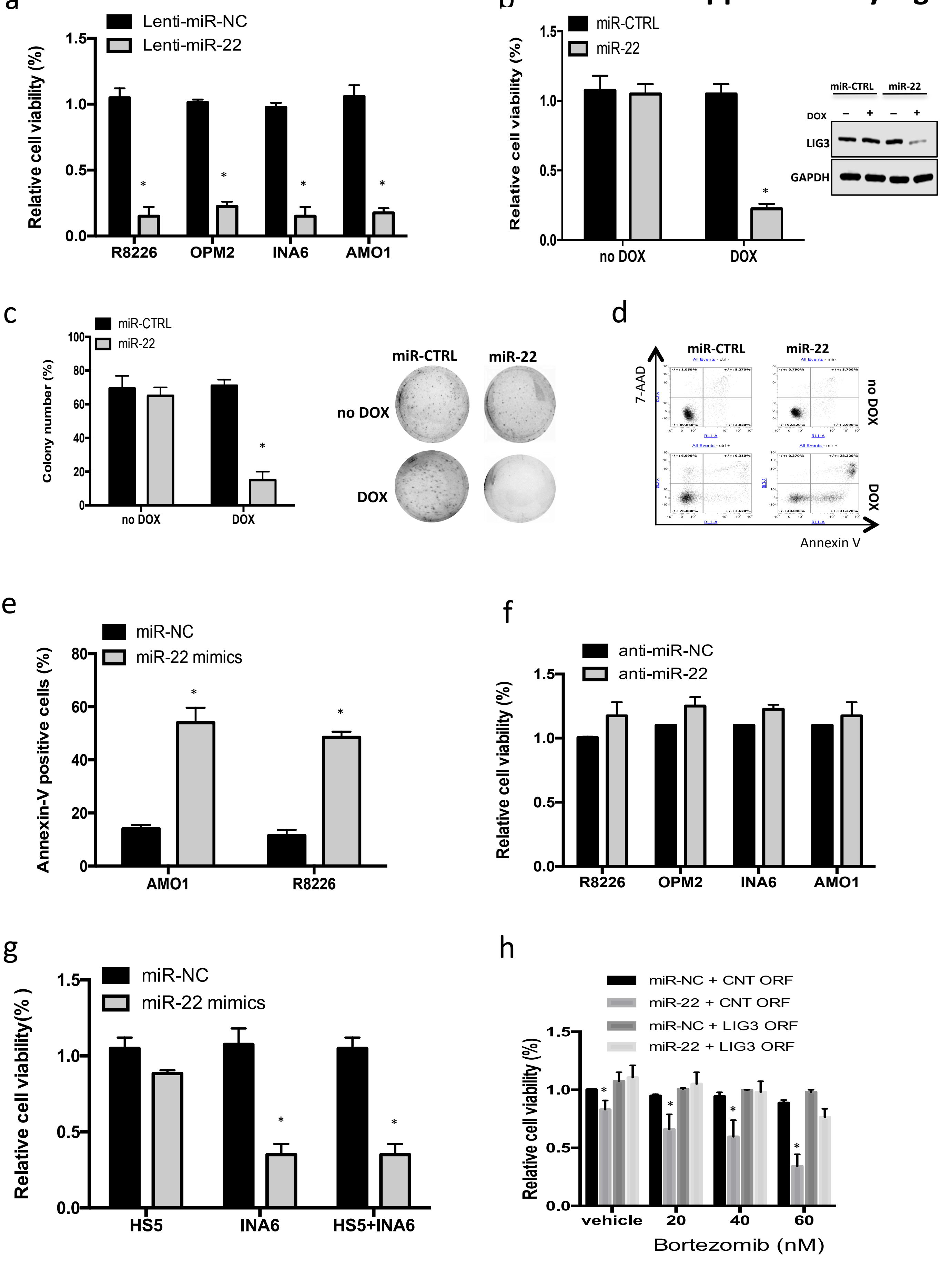

Supplementary Fig.5

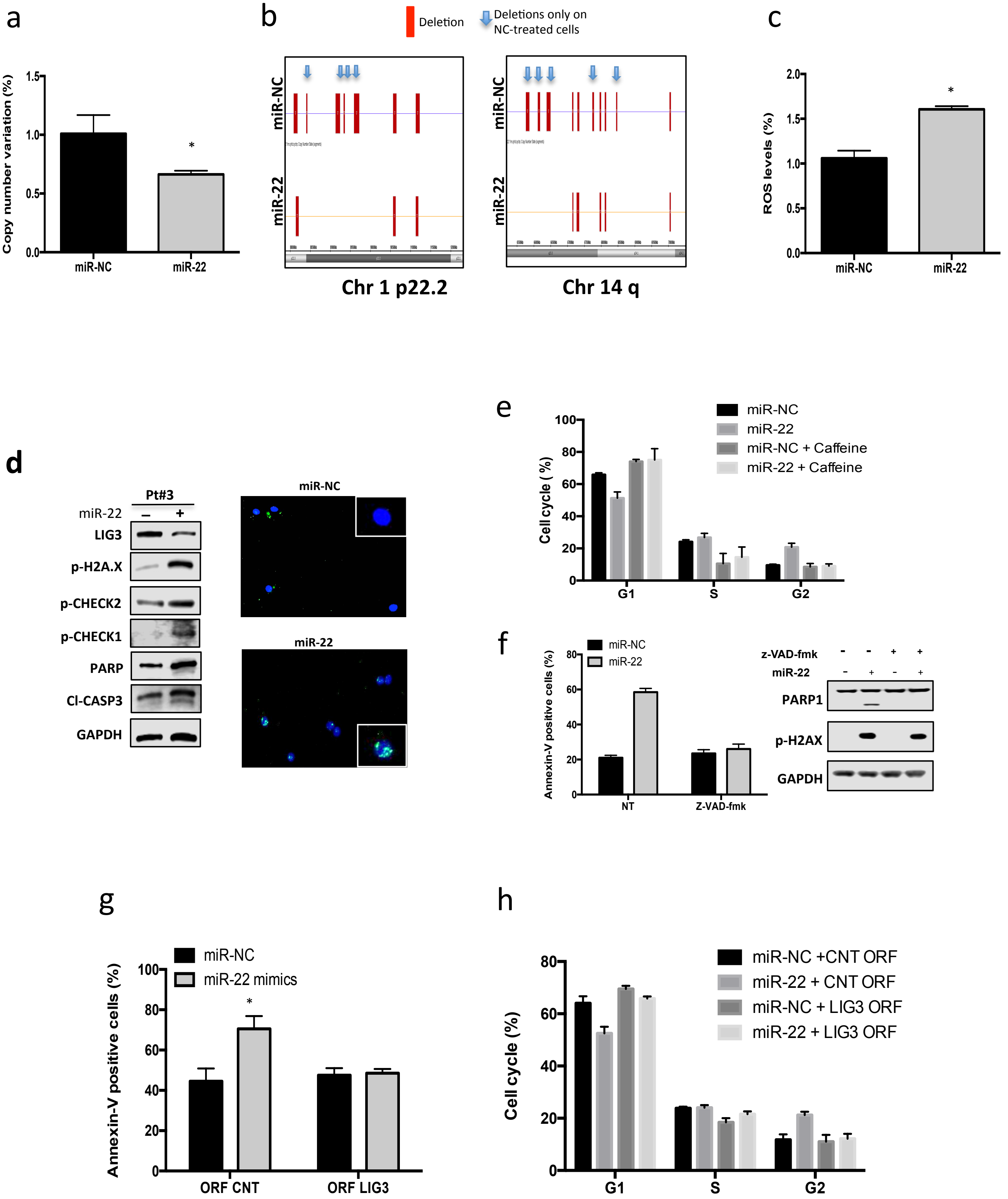

Supplementary Fig.6

a

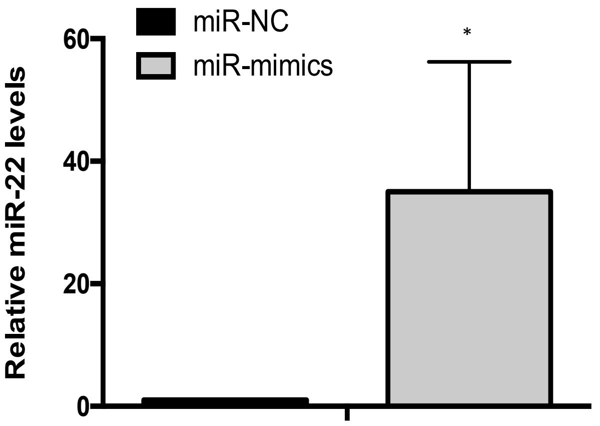

b

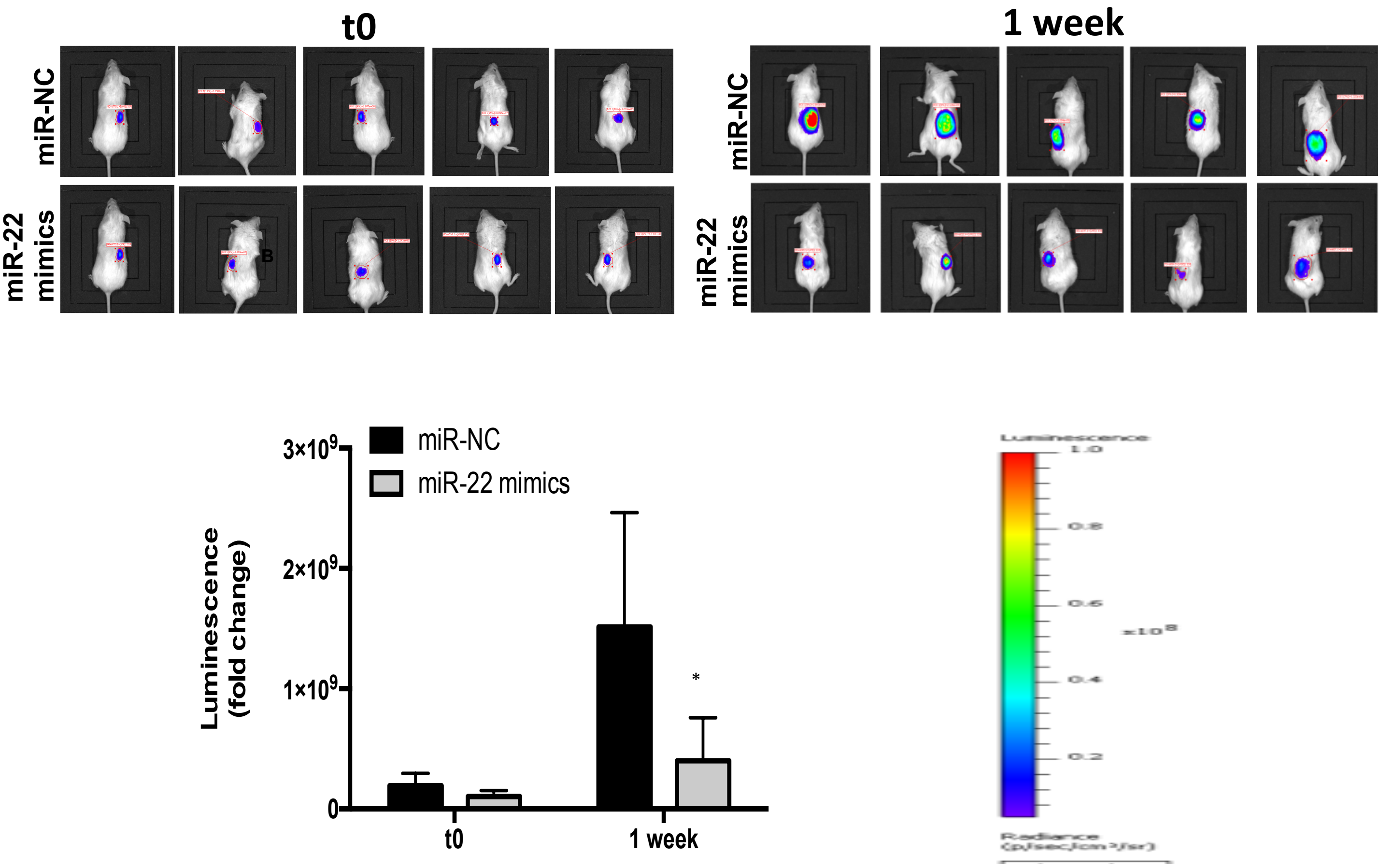

Supplement: Supplementary file 2 — Supplementary Figures [file 41375_2018_238_MOESM2_ESM.pdf]
