# Supplementary material for: miR-22 suppresses DNA ligase III addiction in multiple myeloma
Source: Leukemia. 2018 Aug 17;33(2):487–98. doi: 10.1038/s41375-018-0238-2 (PMC6365379; doi:10.1038/s41375-018-0238-2)
Supplement: Supplementary file 3 — Legends to Supplementary Figures [file 41375_2018_238_MOESM3_ESM.pdf]

Legends to Supplementary Figures

Supplementary Fig.1: (a-b) Data obtained from GSE24080 dataset showing prognostic relevance of LIG1 and LIG4 expression on Overall Survival (a) and Event free Survival (b) of MM patients. **(c)** Analysis of LIG1 and LIG4 mRNA levels in healthy donors and patients' multiple myeloma and plasmacell leukemia cells from GSE39683. *, P<0.001; **, P<0.0001. **(d)** Copy number variation analysis of LIG3 gene in MM patients from MMRF researcher gateway portal (https://research.themmrf.org). **(e)** Analysis of LIG3 mRNA levels according to molecular risk-classification of MM, as evaluated by interrogating GSE19784 dataset. *, P<0.0001. **(f)** LIG3 mRNA levels in MM patients harboring indicated cytogenetic abnormalities as compared with patients that are negative for the same lesions, as result from analysis of proprietary MM dataset. *P < 0.05. **(g)** Analysis of GSE9782 dataset showing LIG1 and LIG4 mRNA levels in MM patients with clinical response or progressive disease after Bortezomib treatment. *P < 0.05. **(h)** Data obtained from GSE9782 dataset showing prognostic relevance of LIG3 mRNA levels on therapy response and Overall Survival of MM patients enrolled to Dexamethasone therapy.

S**upplementary Fig.2: (a)** *Left panel*: Western blot for LIG3 performed on AMO1 cells transduced with 3 different shRNAs and scramble control. GAPDH was used as a loading control. After 4 days of infection, puromycin selected cells (48h, 1,5 μg/ml) were harvested and whole-cell lysates were subjected to immunoblot analysis. *Right panel:* Indicated cell lines were transduced with either scramble control or LIG3-shRNA clone #3: CTG Assay was performed on cell lines transduced with shRNA#3 or scrambled controls after 6 days from infection. LIG3 knockdown was confirmed by western blot analysis 4 days after infection. **(b)** LIG3 downregulation was confirmed by qRT-PCR analysis, 72h after vehicle or IPTG treatment**. (c)** Colony formation of MM cells transduced with either inducible IPTG CTRL-shRNA or LIG3-shRNA lentiviral particles. Light microscopy is shown. Numbers of colonies after 2 weeks (average of 3 independent experiments; *P<0.01) **(d,e,f)** AMO1 and RPMI-8226 cells were transfected with scramble control or LIG3-siRNA clone #2. (d) γ-H2AX foci

evaluation by immunofluorescence 48h after cell transfection. DAPI (blue) was used for nuclear staining. (e) Immunoblot analysis of LIG3, p-H2AX, cleaved form of caspase-3 and PARP. GAPDH was used as a loading control. Analysis was performed 48h after transfection. (f) Annexin V-positive cells 72h after transfection. **(g)** *Left panel:* CTG Assay was performed on AMO-1 and ABZB treated for 24h with increasing dose of Bortezomib. Difference of basal LIG3 expression was evaluated by western blot analysis. *Right panel:* CTG Assay was performed on AMO-1 transfected with LIG3 ORF or control ORF (CNT) and then treated for 24h with increasing dose of Bortezomib. LIG3 overexpression was confirmed by western blot analysis. **(h)** CTG Assay was performed on ABZB transfected with scramble control or LIG3-siRNA clone #2. LIG3 knockdown was confirmed by western blot analysis.

Supplementary Fig.3: (a) Bioinformatic screening of LIG3-targeting miRNAs was performed using mirDIP prediction software. Five miRNAs, were predicted to target LIG3 with excellent score range (≥0.3) in 12 different data sources. **(b)** Graphs of correlations between endogenous mRNA expression levels of LIG3 and mirDIP-selected miRNAs in PCL patients from proprietary dataset. **(c)** qRT-PCR analysis of miR-22-3p expression using total RNA from 3 primary patient MM cells, 5 MM cell lines and 2 samples of bone marrow-derived plasma cells from healthy donors. miR-22-3p levels in PBMC#1 were set as an internal reference. **(d)** *Left panel*: miR-22 levels in MM patients stratified according TC classification; *Right panel*: miR-22 levels in AMO-1 cells transfected with scramble control or MYC-siRNA. **(e)** Schematic representation of LIG3 3'-UTR. The color red indicates the seed sequence of miR-22-3p. **(f)** miR-22 overexpression or inhibition were confirmed by qRT-PCR analysis 48h after miR-22 mimics or inhibitor transfection, respectively. miR-22 levels in miR-NC or anti-miR-NC transfected cells were set as internal reference. *, $P < 0.01$.

Supplementary Fig.4. (a) CTG assay was performed on indicated MM cell lines 6 days after transduction with either miR-NC or miR-22 expressing lentiviral particles. Results are expressed as percentage of NC-transduced cells. *, $P < 0.01$. **(b)** Cell viability was evaluated on AMO1 cells transduced with either inducible-CTRL or inducible-miR-22 expressing lentiviral particles and then treated with

or without doxycycline (500 ng/mL) for 6 days. *, P < 0.01; LIG3 knockdown was confirmed by Western blotting 4 days from doxycycline induction.miR-22 overexpression was confirmed by qRT-PCR analysis 4 day after infection. **(c)** Colony formation of inducible-CTRL or inducible-miR-22 AMO1 cells treated with or without doxycycline (500 ng/mL). Light microscopy is shown. Number of colonies after 2 weeks. **(d)** Annexin V / 7-AAD staining flow cytometry assay of inducible-CTRL or inducible-miR-22 AMO1 cells treated with or without doxycycline (500 ng/mL) for 5 days. **(e)** Annexin V / 7-AAD staining of AMO1 and R8226 cells transfected with miR-22 or miR-NC. **(f)** CTG Assay was performed 96h after transfection of indicated MM cell lines with miR-22 inhibitor or scrambled controls (anti-miR-NC). Results are expressed as percentage of NC-transfected cells. **(g)** CTG Assay was performed 96h after transfection of INA6 cells alone or co-coltured with HS5 cells, with miR-22 mimics or scrambled controls (miR-NC). **(h)** ABZB cells were co-transfected with LIG3 ORF or control ORF and miR-22 mimics or miR-NC and then treated with Bortezomib: CTG survival assay were performed 48h after transfection. *, P < 0.01. Data represent the average ±SD of 3 independent experiments.
Results are representative of three independent experiments ± SD. *, P < 0.01.

Supplementary Fig.5 (a,b) Affymetrix CytoScan HD Array analysis, using genomic DNA from AMO1 overexpressing miR-22 or negative control (miR-NC). **(a)** Acquisition of new copy number variations (CNV) is expressed as percentage of miR-NC cells. **(b)** Representative images of new deletions acquisition on chromosome 1(1p22.2) and chromosome 14 (14q23.3-24.1). Red lines represent deletions. Blue arrows indicate deletions only exiting in miR-NC AMO1 cells. **(c)** ROS-Glo H2O2 Assay performed on AMO1 48 hours after cell transfection. **(d)** Primary plasmacells (Pt #3) were transfected with miR-22 or miR-NC. *Left panel:* Immunoblot analysis was performed after 48h from transfection. *Right panel*: γ-H2AX foci evaluation by immunofluorescence 48 hours after cell transfection. **(e)** AMO1 cells were transfected with miR-22 mimics or miR-NC. 6 hours after electroporation either vehicle or Caffeine (5mM) were added to culture medium. Cell cycle analysis was performed 48h after transfection. **(f)** AMO1 cells were transfected with miR-22 mimics or miR-NC. 6 hours after

electroporation either DMSO (NT) or Z-VAD-FMK were added to culture medium, at final concentration of 25 μM: *Left panel:* Annexin V staining 72 hours after transfection**.** *Right panel*: Immunoblot analysis was performed 48 hours after cell transfection. **(g)** AMO1 cells were co-transfected with LIG3 ORF or control ORF and miR-22 mimics or miR-NC Annexin V/7-AAD staining 72 hours after transfection**. (h)** Cell cycle analysis 48 hours after cell transfection. Results are representative of three independent experiments ± SD. *, $P < 0.05$; **, $P < 0.01$.

Supplementary Fig.6: **(a)** miR-22 overexpression was confirmed by qRT-PCR from a representative AMO1 xenograft per group; miR-22 levels in miR-NC-tumor were set as internal reference. *, $P < 0.01$. **(b)** IVIS images and BLI-based measurement of tumor volumes (5 animals for each group) of NOD SCID mice s.c. xenografted with luciferase gene-marked AMO1, were performed before (t0) and 1 week after treatment.
